# Biosynthesis and Uptake of Copper Nanoparticles by Dead Biomass of *Hypocrea lixii* Isolated from the Metal Mine in the Brazilian Amazon Region

**DOI:** 10.1371/journal.pone.0080519

**Published:** 2013-11-25

**Authors:** Marcia R. Salvadori, Luiz F. Lepre, Rômulo A. Ando, Cláudio A. Oller do Nascimento, Benedito Corrêa

**Affiliations:** 1 Departamento de Microbiologia, Instituto de Ciências Biomédicas II, Universidade de São Paulo, São Paulo, São Paulo, Brazil; 2 Departamento de Química Fundamental, Instituto de Química, Universidade de São Paulo, São Paulo, São Paulo, Brazil; 3 Departamento de Engenharia Química, Politécnica, Universidade de São Paulo, São Paulo, São Paulo, Brazil; University of Houston, United States of America

## Abstract

A biological system for the biosynthesis of nanoparticles (NPs) and uptake of copper from wastewater, using dead biomass of *Hypocrea lixii* was analyzed and described for the first time. The equilibrium and kinetics investigation of the biosorption of copper onto dead, dried and live biomass of fungus were performed as a function of initial metal concentration, pH, temperature, agitation and inoculum volume. The high biosorption capacity was observed for dead biomass, completed within 60 min of contact, at pH 5.0, temperature of 40°C and agitation speed of 150 rpm with a maximum copper biosorption of 19.0 mg g^−1^. The equilibrium data were better described using the Langmuir isotherm and kinetic analysis indicated that copper biosorption follows a pseudo-second-order model. The average size, morphology and location of NPs biosynthesized by the fungus were determined by scanning electron microscopy (SEM), energy dispersive X-ray spectroscopy (EDS) and transmission electron microscopy (TEM). NPs were mainly spherical, with an average size of 24.5 nm, and were synthesized extracellularly. The X-ray diffraction (XRD) analysis confirms the presence of metallic copper particles. Infrared spectroscopy (FTIR) study revealed that the amide groups interact with the particles, which was accountable for the stability of NPs. This method further confirmed the presence of proteins as stabilizing and capping agents surrounding the copper NPs. These studies demonstrate that dead biomass of *Hypocrea lixii* provides an economic and technically feasible option for bioremediation of wastewater and is a potential candidate for industrial-scale production of copper NPs.

## Introduction

Recently, the synthesis of inorganic nanoparticles has been demonstrated by many physical and chemical means. However, the importance of biological synthesis is being emphasized globally at present because chemical methods are capital intensive toxic, non-ecofriendly and have low yield [1]. Copper NPs, due to their unique physical and chemical properties and low cost of preparation, have been of great interest recently. Furthermore, copper NPs have potential industrial use such as gas sensors, catalytic processes, high temperature superconductors, solar cells and so on [2]–[3]. New alternatives for the synthesis of metallic NPs are currently being explored through bacteria, fungi, yeast and plants [4]. Wastewater from copper mines often contain a high concentration of this toxic metal generated during its extraction, beneficiation, and processing. In recent years, bioremediation, through the biosorption of toxic metals as copper has received a great deal of attention, not only as a scientific novelty, but also because of its potential industrial applications. This novel approach is competitive, effective, and cheap [5]. In this respect, fungi have been used in bioremediation processes since they are a versatile group that can adapt to and grow under extreme conditions of pH, temperature and nutrient availability, as well as at high metal concentrations [6]. Consequently, there has been considerable interest in developing biosynthesis methods for the preparation of copper NPs as an alternative to physical and chemical methods. Literature review of previous studies revealed that few articles were published on the biosynthesis of copper NPs [1]. We can mention some examples as Hassan et al.[7], Honary et al. [8], Ramanathan et al. [9], Singh et al. [10], but none of these studies used the fungus *Hypocrea lixii* (*H. lixii*). Also, most of the biosynthesis studies on copper NPs focused on bioreduction phase only, but not the important biosorption phase of the process. Microbial systems have found an important role in NPs production due to their natural mechanism for detoxification of metallic ions through bioreduction, being one of the primary process of biosynthesis, that can be achieved extracellularly, through biosorption [11]. Therefore, the study of biosorption coupled to the process of bioreduction offers advantages as the optimization of physico-chemical parameters (pH, temperature, metal concentration, interaction time and others) that govern the biosorption providing an increase in biosynthesis process efficiency. It also makes it a potential industrially scalable process, and offers a more holistic view of the biosynthesis. In order to enlarge the scope of biological systems for the biosynthesis of metallic nanomaterials and bioremediation of wastewater, we explored for the first time in this work the potential of the fungus *H. lixii*, to the uptake and reduction of copper ions to copper NPs. Thus, the bioremediation and green synthesis of copper NPs has been achieved in the present study using dead biomass of *H. lixii*.

## Materials and Methods

### Ethics Statement

The company Vale S.A., owner of Sossego Mine, located in Canaã, Pará, in the Brazilian Amazon region, through the director of the Vale Technology Institute, Dr Luiz Eugenio Mello authorizing the establishment and dissemination of the study featured in this research article, allowing the collection of material (water from pond of copper waste) supervised by company employees, whose material led to the isolation of the fungus under study. This field study did not involve manipulation of endangered or protected species by a government agency.

### Growth and maintenance of the organism

*H. lixii* was isolated from the water collected from a pond of copper waste from Sossego mine, located in Canãa dos Carajás, Pará, Brazilian Amazonia region (06° 26′ S latitude and 50° 4′ W longitude). *H. lixii* was maintained and activated on Sabouraud Dextrose Agar (SDA) (Oxoid, England) [12].

### Minimum inhibitory concentration in agar medium

Copper tolerance of the isolated fungus was determined as the minimum inhibitory concentration (MIC) by the spot plate method [13]. SDA plates containing different copper concentrations (50 to 2000 mg L^−1^) were prepared and inocula of the tested fungus were spotted onto the metal and control plates (plate without metal). The plates were incubated at 25°C for at least 5 days. The MIC is defined as the lowest concentration of metal that inhibits visible growth of the isolate.

### Studies of the effects of physico-chemical factors on the efficiency of adsorption of copper NPs by the biosorbent

All chemicals used in the present study were of analytical grade and were used without further purification. All dilutions were prepared in double-deionized water (Milli-Q Millipore 18.2 Ωcm^−1^ conductivity). The copper stock solution was prepared by dissolving CuCl_2_.2H_2_O (Carlo Erba, Italy) in double-deionized water. The working solutions were prepared by diluting this stock solution.

The fungal biomass was prepared in Sabouraud broth (Sb) (Oxoid, England), and incubated at 25°C for 5 days, at 150 rpm. After incubation, the pellets were harvested and washed with of double-deionized water this was referred to as live biomass. For the preparation of dead biomass, an appropriate amount of live biomass was autoclaved. The dried biomass was obtained through drying of the fungal mat at 50°C until it became crispy. The dried mat was ground to obtain uniform sized particles.

The pH (2–6), temperature (20–60°C), contact time (5–360 min), initial copper concentration (50–500 mg L^−1^), and agitation rate (50–250 rpm) on the removal of copper was analyzed. Such experiments were optimized at the desired pH, temperature, metal concentration, contact time, agitation rate and biosorbent dose (0.15–1.0 g) using 45 mL of 100 mg L^−1^ of Cu (II) test solution in plastic flask.

Several concentrations of copper (II) were prepared by appropriate dilution of the copper (II) stock solution. The pH was adjusted with HCl or NaOH. The desired biomass dose was then added and the content of the flask was shaken for the desired contact time in an electrically thermostatic reciprocating shaker at the required agitation rate. After shaking, the Cu (II) solution was separated from the biomass by vacuum filtration through a Millipore membrane. The metal concentration in the filtrate was determined by flame atomic absorption spectrophotometer (AAS). The efficiency (R) of metal removal was calculated using the following equation:
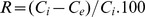
where C_i_ and C_e_ are initial and equilibrium metal concentrations, respectively. The metal uptake capacity, q_e_, was calculated using the following equation:
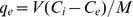
where q_e_ (mg g^−1^) is the biosorption capacity of the biosorbent at any time, M (g) is the biomass dose, and V (L) is the volume of the solution.

### Sorption isotherm and kinetics models

Biosorption was analyzed by the batch equilibrium technique using the following sorbent concentrations of 50–500 mg L^−1^. The equilibrium data were fit using Freundlich and Langmuir isotherm models [14]. The linearized Langmuir isotherm model is:
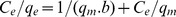
where q_m_ is the monolayer sorption capacity of the sorbent (mg g^−1^), and b is the Langmuir sorption constant (L mg ^−1^). The linearized Freundlich isotherm model is:
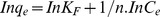
where K_F_ is a constant relating the biosorption capacity and 1/n is related to the adsorption intensity of adsorbent.

The kinetics of Cu (II) biosorption were analyzed using pseudo-first-order, and pseudo-second-order models. The linear pseudo-first-order model [15] can be represented by the following equation:
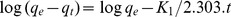
where, q_e_ (mg g^−1^) and q_t_ (mg g^−1^) are the amounts of adsorbed metal on the sorbent at the equilibrium time and at any time t, respectively, and K_1_ (min^−1^) is the rate constant of the pseudo-first-order adsorption process. The linear pseudo-second-order model [16] can be represented by the following equation:
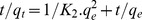
where K_2_ (g mg^−1^ min^−1^) is the equilibrium rate constant of pseudo-second-order.

### Biosynthesis of metallic copper NPs by *H. lixii*

In this study was used only the dead biomass of *H. lixii* that showed a high adsorption capacity of copper metal ion compared to live and dried biomass. Biosynthesis of copper NPs by dead biomass of *H. lixii* was investigated using the data of the equilibrium model at a concentration of 100 mg L^−1^ of copper (II) solution. Analysis by Transmission electron microscopy (TEM) was used for determining the size, shape and location of copper NPs on biosorbent, where cut ultra-thin of the specimens, were observed in a transmission electron microscope (JEOL-1010). X-ray diffraction (XRD) measurements were carried out on a Rigaku Ultima Plus theta-theta diffractometer operated at a voltage of 40 kV and a current of 30 mA with CuK_α_ radiation. Analysis of small fragments of the biological material before and after the formation of copper NPs, was performed on pin stubs and then coated with gold under vacuum and were examined by SEM on a JEOL 6460 LV equipped with an energy dispersive spectrometer (EDS). Infrared vibrational spectroscopy (FTIR) was used to identify the functional groups present in the biomass and to evaluate the spectral variations caused by the presence of copper NPs. The infrared absorption spectra were obtained on Bruker model ALPHA interferometric spectrometer. The samples were placed directly into the sample compartment using an attenuated total reflectance accessory of single reflection (ATR with Platinium-crystal diamond). Eighty spectra were accumulated for each sample, using spectral resolution of 4 cm^−1^.

## Results and Discussion

*H. lixii*, isolated from copper mine, was subjected to minimum inhibitory concentration (MIC) at different copper concentrations (50–2000 mg L^−1^) and the results indicated that *H. lixii* exhibited high tolerance to copper (528 mg L^−1^). The fungal cell wall is mainly composed of polysaccharides, some of them bound to proteins, and other components such as lipids and melanins [17]. Such biomolecules on the fungal cell wall components have various functional groups such as amino, carboxyl, thiol, sulphydryl and phosphate groups which play an important role in the sorption of various metals [18].

### Influence of the physico-chemical factors on biosorption

**Figure 1:**
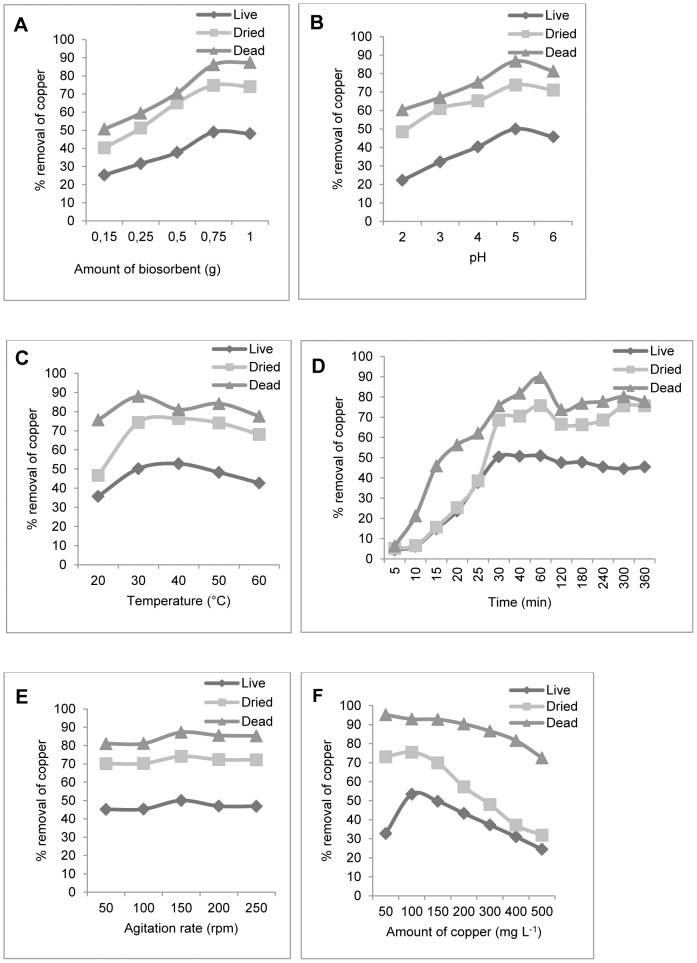
Batch biosorption studies. Influence of the physico-chemical factors on the live, dried and dead biomass of *H. lixii*. (A) Effect of the amount of biosorbent. (B) Effect of pH. (C) Effect of temperature. (D) Effect of contact time. (E) Effect of agitation rate. (F) Effect of initial copper concentration.

The present investigation showed that copper removal by *H. lixii* biomass was influenced by physico-chemical factors such as biomass dosage, pH, temperature, contact time, rate of agitation and metal ion concentration. The biosorbent dose is an important parameter since it determines the adsorption capacity of a biosorbent for a given initial concentration of the metals. As observed in Figure 1A, the removal of cooper by live and dried biomass of *H. lixii* increased with increasing biomass concentration and reached saturation at 0.75 g, whereas the saturation was reached to 1.0 g for dead biomass (Figure 1A). The percent removal of copper by dead biomass was greater than that observed for live and dried biomass (Figure 1A). The dead biomass for Cu (II) removal offers the following advantages: the metal removal system is not subjected to the toxic effect of the same, it does not require growth media and adsorbed metal ions can be easily desorbed and dead biomass can be reutilized. Copper removal by live and dried biomass decreased with an increase of biomass concentration beyond 0.75 g L^−1^. This finding indicates that dead biomass possess a higher affinity for copper than live and dried biomass. The increase in removal capacity with increasing biomass dose can be attributed to a greater total surface area and a consequent larger number of binding sites. Maximum removal of copper was observed at pH 5.0 for the three types of biomass as shown in Figure 1B. At lower pH value, the cell wall of *H. lixii* becomes positively charged and it is responsible for reduction in biosorption capacity. In contrast, at higher pH (pH 5), the cell wall surface becomes more negatively charged and therefore the biosorption of Cu (II) onto *H. lixii* is high due to attraction between the biomass and the positively charged metal ion. Some researchers have also investigated the effect of pH on the biosorption of toxic metals and found similar results [19]–[20]. The maximum removal of copper was observed at 40°C for the three types of biomass (Figure 1C). The effect of temperature on biosorption of the metal suggests an interaction between the metal and the ligands on the cell wall. It is observed that the graph (Figure 1D) follows the sigmoid kinetics which is characteristic of enzyme catalyzed reaction for the three types of biomass. The kinetics of copper NPs formation by dead biomass showed that more than 89% of the particles were formed within the 60 min of the reaction, which suggests that the formation of copper NPs is exponential. The optimum copper removal was observed at an agitation speed of 150 rpm for the three types of biomass (Figure 1E). At high agitation speeds, vortex phenomena occur and the suspension is no longer homogenous, a fact impairing metal removal [21]. The percentage of copper adsorption decreased with increasing metal concentration (50–500 mg L^−1^) for the three types of biomass as shown in Figure 1F. The same has been observed for fungi at concentration of Zn ranging from 100–400 mg L^−1^ [22].

### Sorption isotherm and kinetics models

**Figure 2:**
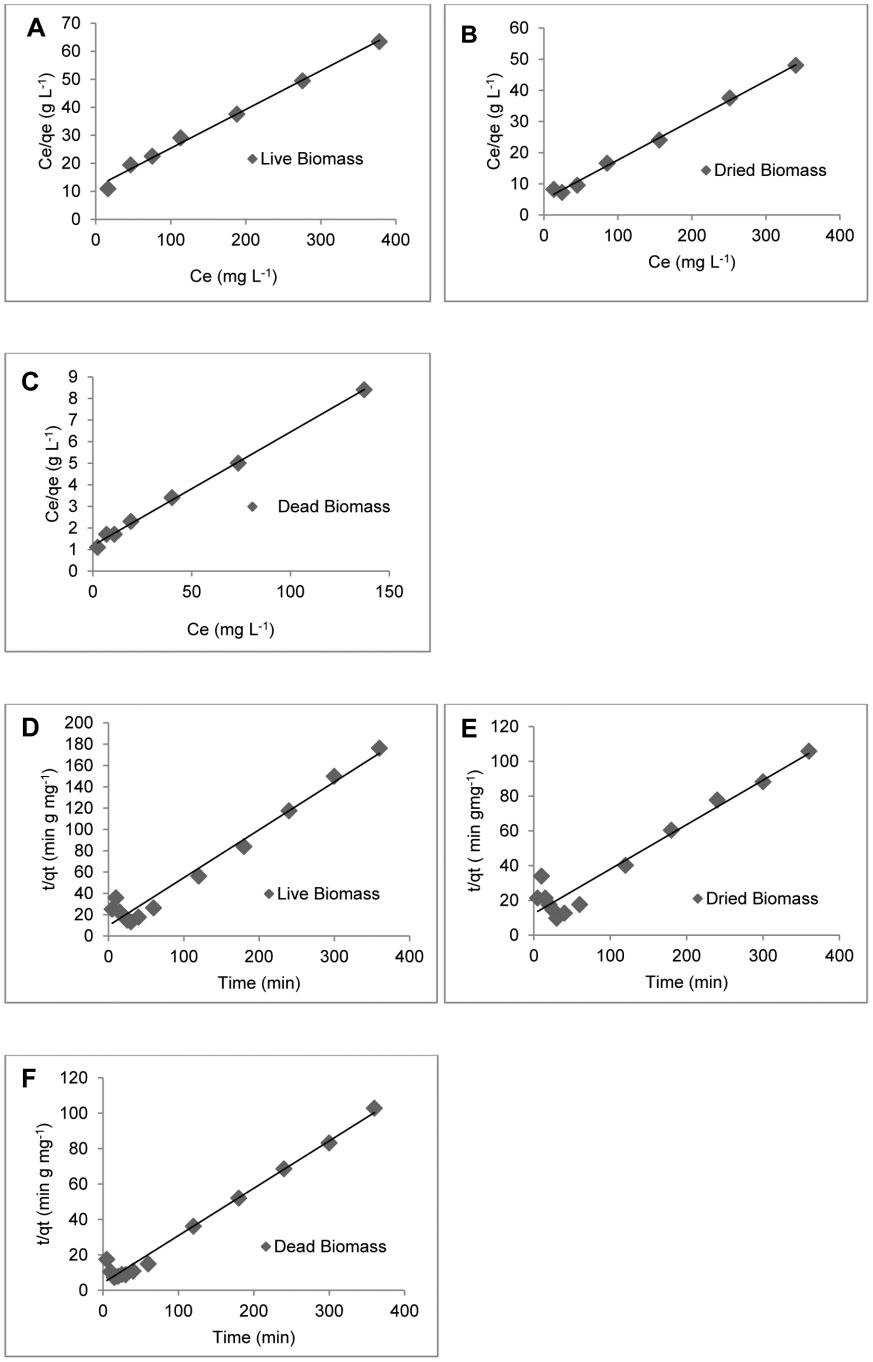
Biosorption isotherm models and biosorption kinetics of *H. lixii*. Langmuir plots for live (A), dried (B) and dead (C) biomass. Pseudo second-order models for live (D), dried (E) and dead (F) biomass.

The Langmuir and Freundlich isotherm models were used to fit the biosorption data and to determine the biosorption capacity. The Langmuir isotherm for Cu (II) biosorption obtained for the three types of *H. lixii* biomass is shown in Figure 2A, Figure 2B and Figure 2C. The isotherm constants, maximum loading capacity estimated by the Langmuir and Freundlich models, and regression coefficients are shown in Table 1. The Langmuir model better described the Cu (II) biosorption isotherms than the Freundlich model. The maximum adsorption rate of Cu (II) by *H. lixii* (19.0 mg g^−1^) observed in this study was similar to or higher than those reported for other known biosorbents, such as *Pleurotus pulmonaris*, *Schizophyllum commune*, *Penicillium spp* and *Rhizopus arrhizus*, with adsorption rates of 6.2, 1.52, 15.08 and 19.0 mg g^−1^ respectively [23–24–25]. The kinetics of Cu (II) biosorption onto the three types of biomass of *H. lixii* were analyzed using pseudo-first-order and pseudo-second-order models. All the constants and regression coefficients are shown in Table 2. In the present study, biosorption by *H. lixii* was best described using a pseudo-second-order kinetic model as shown in Figure 2D, Figure 2E and Figure 2F. This adsorption kinetics is typical of the adsorption of divalent metals onto biosorbents [26].

**Table 1:** Adsorption constants from simulations with Langmuir and Freundlich models.

	Langmuir model			Freundlich model		
Type of biomass	q_m_(mg g^−1^)	b(L mg^−1^)	*R^2^*	K_F_(mg g^−1^)	1/n	*R^2^*
Live	7.2	0.012	0.993	0.44	0.44	0.972
Dried	8.0	0.025	0.995	0.59	0.39	0.857
Dead	19.0	0.044	0.997	1.37	0.51	0.966

**Table 2:** Kinetic parameters for adsorption of copper.

	Pseudo-first-order		Pseudo-second-order	
Type of biomass	K_1_(min^−1^)	*R^2^*	K_2_(g mg^−1^min^−1^)	*R^2^*
Live	2.30×10^−3^	0.026	19.78×10^−3^	0.968
Dried	1.51×10^−2^	0.774	6.95×10^−3^	0.936
Dead	3.91×10^−3^	0.404	14.82×10^−3^	0.982

### Biosynthesis of copper NPs

**Figure 3:**
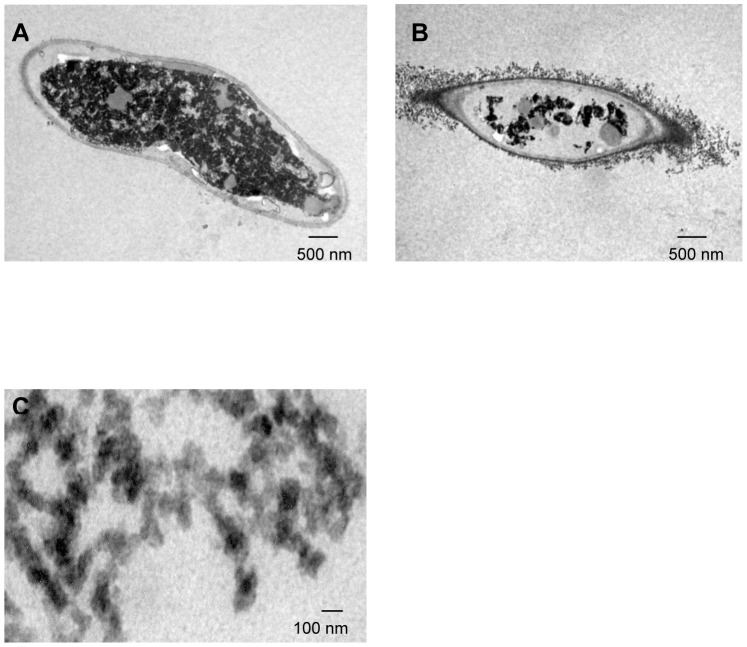
TEM micrographs of *H. lixii* sections. (A) Control (without copper), (B) Section of the fungus showing extracellular localization of copper nanoparticles and (C) Copper nanoparticles.

**Figure 4:**
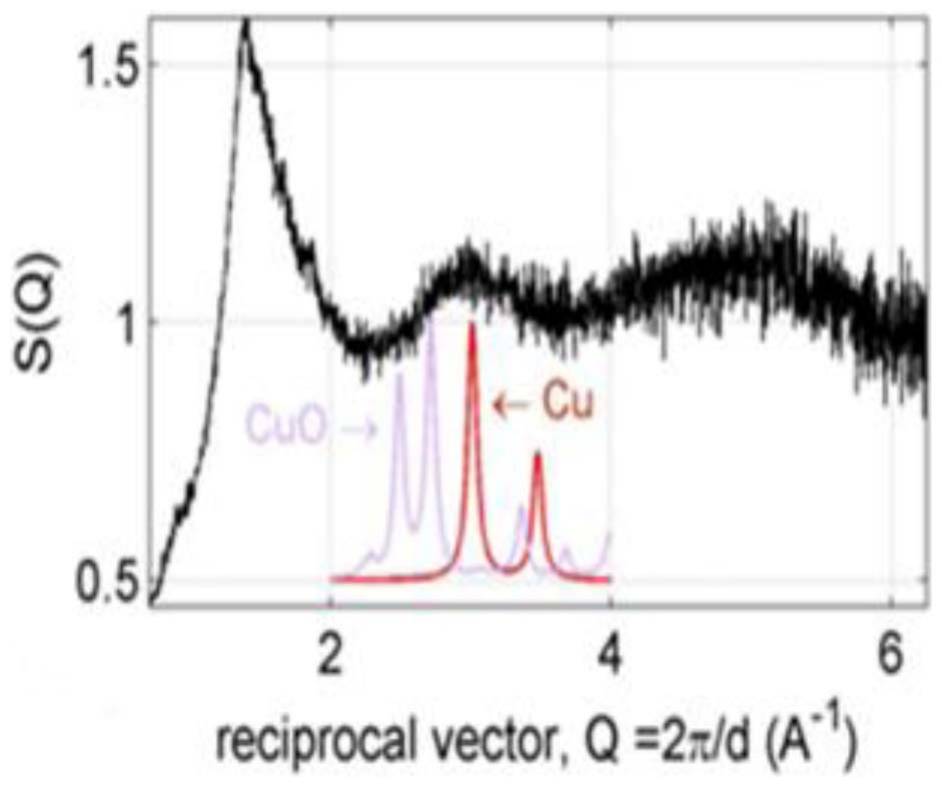
XRD analysis. Structural function S (Q) compared to the XRD patterns of the metallic copper and CuO.

**Figure 5:**
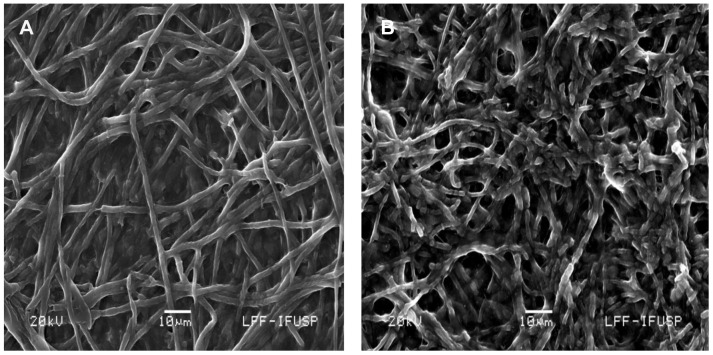
Dead biomass of *H. lixii* analyzed by SEM-EDS. (A) Control (without copper) and (B) biomass exposed to copper.

**Figure 6:**
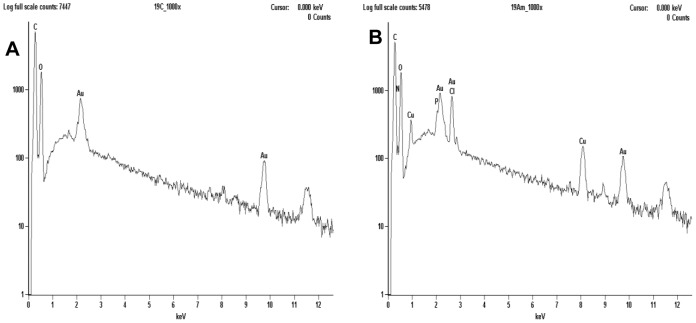
EDS spectra recorded of dead biomass of *H. lixii*. (A) before exposure to copper solution and (B) after exposure to copper

**Figure 7:**
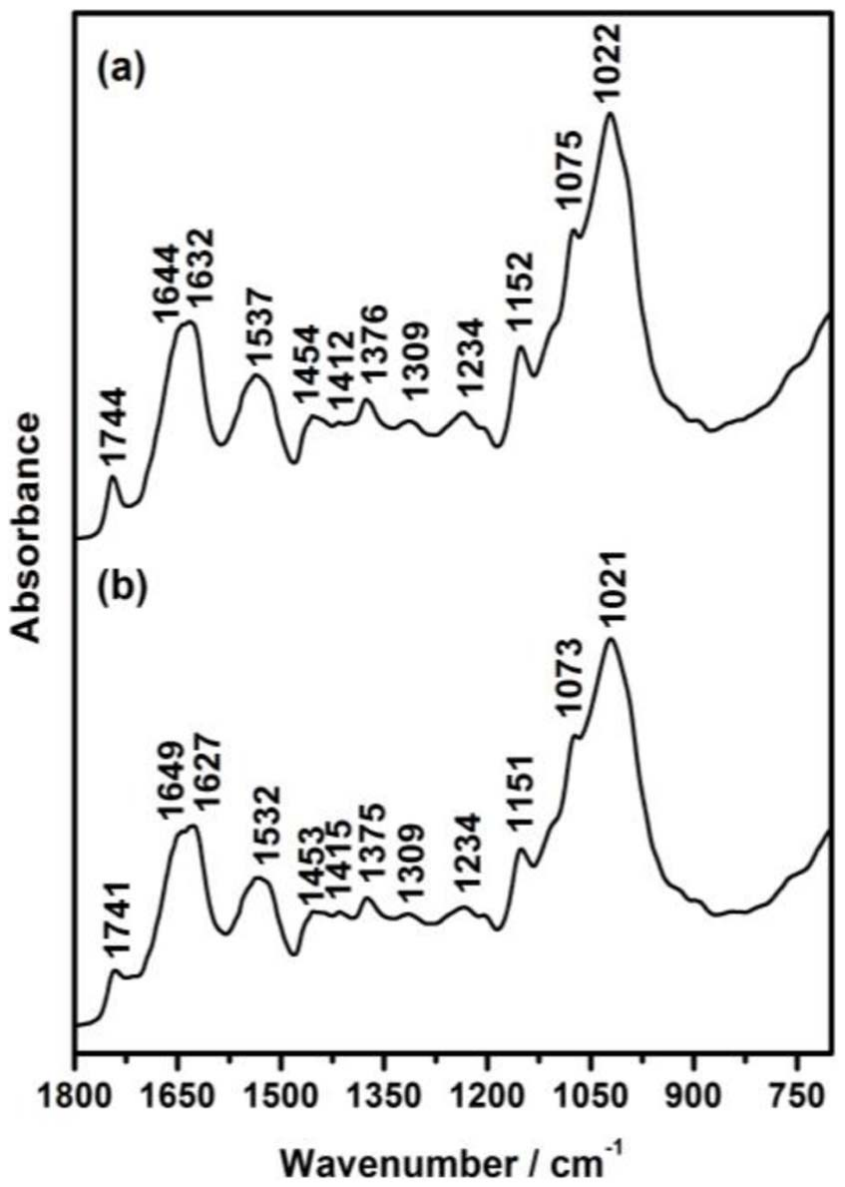
FTIR spectra of dead biomass of *H. lixii*. (A) before and (B) after to saturation with copper ions.

The studying of the involved mechanisms of the NPs formation by biological systems is important in order to determine even more reliable and reproducible methods for their biosynthesis. To understanding the formation of NPs in fungal biomass, was examined by TEM a fraction of the dead biomass. The location of the NPs in *H. lixii* was investigated and the electron micrograph revealed that NPs were found in the cell wall, but not in cytoplasm and cytoplasmic membrane, and was absent in control, the ultrastructural change such as shrinking of cytoplasmatic material was observed in control and biomass impregnated with copper, which was due to autoclaving process (Figure 3A and Figure 3B). The extracellular location, offers the advantages of obtaining NPs faster and in large amounts, easy removal and possible reuse of the biomass in the production process. The shape and size of NPs are important features controlling the physical, chemical, optical and electronic properties of the nanoscopic materials [27]–[28]. In this study copper NPs showed an average diameter of 24.5 nm. At magnifications 100 nm, the particles are predominantly spherical as shown in Figure 3C. Comparison of the XRD patterns of metallic Cu and CuO shown in Figure 4 clearly shows that the broad peak of *Q* = 3Å^−1^ (or 2θ≈40°) is centered at the position of the main diffraction peak of metallic Cu, (reflection 111, *d* = 2.09Å), indicating that the NPs synthesized by dead biomass of *H. lixii* are in fact NPs of metallic copper. The presence of copper NPs was confirmed by spot profile SEM-EDS measurement. SEM micrographs recorded before and after biosorption of Cu (II) by fungal biomass are shown in Figure 5A and Figure 5B respectively. We observed that a surface modification occurred by increasing the irregularity, after binding of copper NPs onto the surface of the fungus biomass. EDS spectra recorded in the examined region of the mycelium, show signals from copper (Figure 6A and Figure 6B) for the fungus. Apart from this, the signals for C, N and O indicate the presence of proteins as a capping material on the surface of the copper NPs. Such signals are likely to be due to proteins secreted by the fungus, and is supported by FTIR-ATR measurement for the formation of copper NPs, which identified possible interactions between copper and bioactive molecules, that could be responsible for the synthesis and stabilization (capping material) of copper NPs. The amide linkages between amino acid residues in proteins give rise to well know signatures in the infrared region of the electro-magnetic spectrum. FTIR spectrum reveals two bands at 1649 and 1532 cm^−1^, that correspond to the bending vibrations of amide I and amide II, respectively (Figure 7). Such modes arise from peptides/proteins bound to copper NPs, which suggests the possibility of these agents acting as capping agents [29]. In this study, after saturating the biomass samples with copper (II) ions, several bands shifts were observed in the FT-IR spectra in relation to pure samples, especially those assigned to amide groups. The bands at 1644, 1632 and 1537 cm^−1^ were shifted to 1649, 1627 and 1532 cm^−1^, respectively (Figure 7). It suggests that biosorption is due to the interaction between copper ions and amide groups within the available biomass. The two bands observed at 1375 and 1073 cm^−1^ can be assigned to the C-N stretching vibrations of the aromatic and aliphatic amines, respectively (Figure 7) [30]. Such observations indicate the presence and binding of proteins to copper NPs which can lead to their possible stabilization. In dead biomass probably the protein from the cell is liberated during the autoclaving process and bound on the surface cell. This observation indicates that the copper NPs in spherical morphology are present with proteins that are possibly bound to the surface of the NPs thereby acting as stabilizing agents of the spherical NPs. FTIR results obtained during the present study also revealed that amide groups from proteins have strong affinity to bind metals. However the type of protein involved in interactions with NPs of copper which was studied remains to be determined. This knowledge may permit the development of more efficient process for the green synthesis of copper NPs.

## Conclusions

In this work we explored for the first time the extracellular biosynthesis and uptake of copper NPs from wastewater using dead biomass of the filamentous fungus *H. lixii*. In this work was developed a synthetic strategy for the biosynthesis and removal of copper NPs which is fast, low cost, environment friendly and easily scalable, using as a reduction agent the fungus *H. lixii*. In future studies we intend to characterize the biomacromolecules involved in the biosorption and bioreduction mechanisms of copper NPs synthesis.
